# Heart Failure Management in the Modern Era: A Comprehensive Review on Medical and Device-based Interventions

**DOI:** 10.2174/011573403X338702250226075044

**Published:** 2025-03-13

**Authors:** Aman Shrivastava, Niharika Gokhale, Pritesh Paliwal, Sumeet Dwivedi, Shamim Khan, Preetam L. Nikam, Yash Jain, Jaya Kumari

**Affiliations:** 1 Department of Pharmacology, College of Pharmacy, Institute of Professional Studies, Gwalior, Madhya Pradesh, India;; 2 Faculty of Pharmacy, Medi-Caps University, Indore, Madhya Pradesh, India;; 3 Department of Pharmacy, Indore Institute of Pharmacy, Indore, Madhya Pradesh, India;; 4 Department of Pharmacy, Acropolis Institute of Pharmaceutical Education and Research, Indore, Madhya Pradesh, India;; 5 IIMT College of Medical Sciences, IIMT University, Ganga Nagar, Meerut, Uttar Pradesh, India;; 6 Department of Pharmacy, SND College of Pharmacy, Nashik, Maharashtra, India;; 7 Department of Pharmacy Practice, NIMS Institute of Pharmacy, NIMS University, Jaipur, Rajasthan, India

**Keywords:** Heart failure, medical therapy, implantable devices, synergetic potential, cardiovascular disorders, etiology

## Abstract

Heart failure remains a significant global health challenge, necessitating continuous advancements in management strategies to improve patient outcomes. This review aimed to elucidate the current scenario of heart failure and its management in the modern era, focusing on integrating medical therapy and implantable device interventions. According to guidelines, medical treatment remains the primary method of treating heart failure. Such medications include ACE inhibitors, neprilysin-angiotensin receptor inhibitors, beta-blockers, angiotensin II receptor blockers, mineralocorticoid receptor antagonists, and blockers of sodium-glucose co-transporter-2. These pharmacologic agents have demonstrated efficacy in decreasing mortality and morbidity in patients. The advent of implantable devices has revolutionized treatment, providing substantial benefits in specific patient populations. Cardiac resynchronization therapy has emerged as a pivotal intervention for patients with reduced ejection fraction and dyssynchronous ventricular contraction, effectively enhancing cardiac function and quality of life. Furthermore, left bundle branch area pacing improvements provide fascinating alternatives to traditional cardiac resynchronization therapy. The essential significance of device-based therapies is further highlighted by the function of implanted cardioverter-defibrillators in preventing unexpected cardiac deaths in high-risk patients. Furthermore, integrating remote monitoring technologies and novel device innovations continues to enhance the precision and efficacy of heart failure management. This review comprehensively examines current guidelines and evidence supporting the use of these therapies, addressing their synergistic potential and the practical considerations for their implementation, while synthesizing recent advancements in pharmacologic and device-based interventions.

## 1 INTRODUCTION

Heart failure's (HF) complicated etiology and high prevalence of hospitalization and death make it a major and increasing public health concern [1]. Being one of the most prevalent causes of hospitalization and medical expenses worldwide, heart failure poses a significant burden on individuals, healthcare providers, and societies [2]. The medical condition results from structural or functional cardiac problems that affect the capacity of the heart to pump blood effectively, which in turn impacts blood flow to tissues and organs. This often leads to symptoms that adversely impact the quality of life, like exhaustion, retention of water, and dyspnoea [3]. In recent years, advancements in both medical therapies and device-based interventions have revolutionized HF management, offering new hope for improved patient outcomes [4]. Traditional pharmacological treatments, including ACE inhibitors, beta-blockers, and diuretics, have long been the cornerstone of HF therapy, providing symptomatic relief and mortality benefits [5]. However, the heterogeneity of HF, encompassing preserved and reduced ejection fraction phenotypes, necessitates a more tailored approach to treatment [6]. The modern era has witnessed the emergence of novel pharmacological agents, such as angiotensin receptor-neprilysin inhibitors (ARNIs), sodium-glucose co-transporter-2 (SGLT2) inhibitors, and selective sinus node inhibitors, which have demonstrated significant clinical benefits in large-scale trials [7]. Additionally, for patients with severe heart failure or those who are unresponsive to medical treatment, improvements in device-based therapies, such as implantable heart defibrillators (ICDs), cardiac resynchronization therapy (CRT), and ventricular assist devices (VADs), have offered efficient alternatives, as shown in Fig. (**1**) [8, 9]. This comprehensive review aimed to provide an in-depth analysis of the current landscape of HF management, highlighting the latest advancements in medical and device-based interventions [10]. By exploring the efficacy, safety, and clinical implications of these treatments, we seek to offer a valuable resource for healthcare professionals striving to optimize care for patients with HF in the modern era [11]. Through a thorough understanding of these interventions, clinicians can better navigate the complexities of HF treatment, ultimately enhancing patient outcomes and quality of life [12].

## 2 EPIDEMIOLOGY: CURRENT SCENARIO OF HF

HF presents a critical challenge worldwide, with its prevalence and impact growing at an alarming rate. Currently, over 64 million people globally are affected by HF, highlighting the urgent need for effective management strategies [13]. This section provides an overview of the current scenario of HF, utilizing percentage data to illustrate its impact across various demographics and regions [14]. The current scenario of HF underscores the importance of comprehensive public health strategies aimed at prevention, early detection, and effective management. Addressing the disparities in care and ensuring equitable access to advanced HF therapies are crucial for improving patient outcomes and reducing the global burden of this debilitating condition, as shown in Fig. (**2**) [15].

### 2.1 Prevalence and Incidence

Approximately 1% to 2% of the global adult population is affected by HF, with the prevalence increasing to over 10% among individuals aged 70 years and older [16]. In high-income countries, the prevalence of HF ranges from 1% to 2% in the general adult population. For example, in the United States, about 6.2 million adults have HF, constituting roughly 2% of the adult population [17]. These regions are witnessing a rapid rise in HF cases due to increasing rates of hypertension, diabetes, and obesity. The prevalence in these countries can range from 1% to 3%, with significant regional variations [18].

### 2.2 Gender Differences

Males are more likely to be diagnosed with HF with reduced ejection fraction (HFrEF), whereas women are less prone to experience HF with preserved ejection fraction (HFpEF). Among HF patients, approximately 50% to 60% of men have HFrEF, whereas 40% to 50% of women have HFpEF [19]. The prevalence of HF markedly increases with age. About 10% to 20% of individuals aged 70 and older have HF, compared to less than 1% in those aged 50 to 59 years [20]. While HF is less common in younger adults, it still affects around 0.1% to 0.2% of individuals aged 20 to 39 years, often due to congenital heart disease, myocarditis, or genetic cardiomyopathies [21].

### 2.3 Hospitalization and Mortality Rates

HF is a leading cause of hospitalization among older adults. In the United States, HF accounts for over 1 million hospitalizations annually, with readmission rates within 30 days approaching 25% [22]. Despite advances in treatment, the five-year mortality rate for HF patients remains high, at approximately 50%. In-hospital mortality rates for acute HF range from 4% to 10%, varying based on the severity of the condition and comorbidities [23].

### 2.4 Ethnic and Socioeconomic Disparities

African Americans have a 20% greater incidence of heart failure than Caucasians and they are more likely to get the disease at younger ages. Furthermore, the prevalence of HF is rising among South Asians, in part because of greater rates of both diabetes and coronary artery disease [24]. A greater prevalence of heart failure has been associated with lower levels of socioeconomic status. Individuals from low-income backgrounds are more likely to experience barriers to accessing healthcare, leading to delayed diagnosis and suboptimal management [25]. HF imposes significant economic burdens on healthcare systems. In countries with high incomes, like the United States, the yearly cost of HF exceeds $30 billion, encompassing immediate healthcare expenses as well as additional expenses [26]. In low- and middle-income countries, the economic impact is substantial relative to their healthcare budgets, with HF management costs constituting a significant portion of their limited resources [27].

## 3 CURRENT MANAGEMENT GUIDELINES FOR HF

Recent management guidelines for HF reflect advancements in clinical understanding and therapeutic strategies aimed at improving patient outcomes and quality of life [28]. These guidelines emphasize a comprehensive approach encompassing pharmacological, non-pharmacological, and device-based therapies tailored to individual patient needs and characteristics [29]. By using ACE inhibitors, angiotensin receptor blockers, beta-blockers, and mineralocorticoid receptor antagonists, pharmacological therapies aim to optimize neurohormonal inhibition. These medications are crucial in attenuating the pathophysiological processes underlying HF, reducing symptoms, preventing disease progression, and enhancing survival rates. Non-pharmacological management strategies include lifestyle modifications, such as dietary sodium restriction, fluid management, regular physical activity, and smoking cessation [30]. Patient education plays a pivotal role in promoting adherence to these lifestyle changes, which are essential for managing comorbidities and optimizing overall cardiovascular health [31]. Device-based therapies, such as CRT and ICDs, are recommended in specific patient populations to improve cardiac function, alleviate symptoms, and reduce the risk of sudden cardiac death [32]. These interventions are tailored based on individual clinical profiles and responses to initial therapies. Furthermore, recent guidelines underscore the importance of integrated care models involving multidisciplinary healthcare teams, including cardiologists, nurses, pharmacists, and allied health professionals [33]. Collaborative efforts ensure comprehensive assessment, personalized treatment plans, and ongoing monitoring to achieve optimal outcomes in HF management. Recent guidelines for HF advocate for a patient-centered approach that integrates evidence-based pharmacological therapies, lifestyle modifications, and advanced device-based interventions [34]. By addressing individual patient needs and optimizing treatment strategies, these guidelines aim to improve clinical outcomes, enhance quality of life, and reduce hospitalizations associated with HF (Table **1**) [35-40].

## 4 RECENT NOVEL THERAPEUTIC APPROACHES FOR HF MANAGEMENT

HF management has introduced novel therapeutic approaches aimed at improving patient outcomes and addressing the complex nature of the condition [41]. These innovative strategies encompass a range of pharmacological, device-based, and lifestyle interventions that complement traditional treatments, offering new hope for patients with HF [42-50] (Table **2**).

### 4.1 Angiotensin Receptor-Neprilysin Inhibitors

Angiotensin receptor-neprilysin inhibitors, such as sacubitril/valsartan, represent a significant advancement in HF treatment [51]. Angiotensin receptor inhibition and neprilysin inhibition work together to improve heart disease outcomes, including lowering mortality and hospitalization rates, by raising natriuretic peptide levels [52].

### 4.2 Sodium-glucose Co-transporter 2 (SGLT2) Inhibitors

SGLT2 inhibitors, originally developed for diabetes management, have shown promising results in HF treatment [53]. These medications reduce the reabsorption of glucose and sodium in the kidneys, leading to improved cardiac function, reduced hospitalizations due to HF exacerbations, and potentially lower mortality rates, as shown in Fig. (**3**) [54].

### 4.3 Device-based Therapies

Advanced device-based therapies, such as CRT and ICDs, continue to evolve. CRT optimizes ventricular synchrony, while ICDs detect and treat life-threatening arrhythmias, thereby improving heart function and preventing sudden cardiac death in selected patient populations [55]. Some examples are given below:

#### 4.3.1 Conduction System Pacing (CSP)

His bundle pacing (HBP) and left bundle branch pacing (LBBP) are the two forms of CSP, which is a significant advancement in cardiac resynchronization treatment. Unlike traditional pacing methods, CSP preserves the natural conduction pathway, ensuring physiological ventricular activation. It has demonstrated significant improvements in left ventricular function and clinical outcomes, especially in HF patients with conduction abnormalities.

#### 4.3.2 Extravenous Implantable Cardioverter-defibrillator (EV-ICD)

The EV-ICD is a novel device that offers an entirely extravascular approach to defibrillation and pacing. Positioned outside the venous system, it eliminates risks associated with transvenous leads, such as thrombosis and infection. Early studies indicate comparable efficacy to traditional ICDs, making it a promising option for HF patients with high infection risk.

#### 4.3.3 Subcutaneous Implantable Cardioverter-defibrillator (S-ICD)

S-ICD is another innovative device for preventing sudden cardiac death. Unlike conventional ICDs, it avoids direct vascular access, thus minimizing the risk of related complications. Although S-ICDs do not provide pacing capabilities, they are ideal for patients requiring defibrillation-only support [56].

### 4.4 Precision Medicine and Biomarker-guided Therapy

Precision medicine approaches tailor HF treatment based on individual patient characteristics, genetic profiles, and biomarker levels. This personalized approach helps optimize therapy effectiveness, minimize adverse effects, and improve overall patient outcomes [57].

#### 4.4.1 Nonsteroidal Mineralocorticoid Receptor Antagonists (MRA)

Nonsteroidal MRAs, such as finerenone, have emerged as a safer alternative to traditional steroidal MRAs, like spironolactone and eplerenone. They offer effective control of hyperkalemia and are particularly beneficial in HF patients with coexisting chronic kidney disease (CKD). By targeting mineralocorticoid receptors more selectively, they reduce fibrosis and inflammation, lowering the risk of cardiovascular morbidity and mortality.

#### 4.4.2 GLP-1 Receptor Agonists (GLP-1 RA)

Initially developed for managing type 2 diabetes, GLP-1 RAs (*e.g*., liraglutide, semaglutide) have shown cardioprotective benefits in HF patients. They improve glycemic control, enhance weight loss, and reduce inflammation, contributing to better heart function. Moreover, ongoing trials suggest their potential role in reducing HF hospitalization rates.

#### 4.4.3 Dual Agonists (GIP-GLP-1 RA)

Dual receptor agonists, such as tirzepatide, simultaneously target the glucose-dependent insulinotropic polypeptide (GIP) and glucagon-like peptide-1 (GLP-1) pathways. This dual action improves glucose metabolism and insulin sensitivity, with preliminary evidence indicating a positive impact on cardiac remodeling and diastolic function [58].

#### 4.4.4 Lifestyle Interventions and Patient Education

Comprehensive HF management includes lifestyle modifications, such as sodium restriction, fluid management, regular physical activity, and smoking cessation [59]. Patient education plays a crucial role in promoting adherence to prescribed therapies and empowering patients to manage their condition effectively [60].

#### 4.4.5 Traditional Ayurvedic Medicines

Various traditional herbal drugs are used in the treatment of HF [61]. Ayurvedic medicines often focus on supporting heart function through antioxidant, anti-inflammatory, and cardioprotective effects [62]. While some herbs, like Arjuna and garlic, have shown promising effects in clinical studies, others, like *Terminalia chebula* and *Amla*, are traditionally used for their broader health benefits with limited specific evidence in HF [63, 64]. Integrating these medicines with conventional therapies may offer complementary support in managing heart health (Table **3**).

## 5 NOVEL PROSPECTS FOR CARDIAC RESYNCHRONIZATION THERAPY

CRT has emerged as a pivotal intervention in the management of HF, particularly for patients with reduced left ventricular ejection fraction and electrical desynchrony [71]. Recent advancements and ongoing research have unveiled new opportunities to enhance the efficacy and expand the indications of CRT, offering hope for improved outcomes among HF patients [72].

### 5.1 Enhanced Device Technology

Modern CRT devices incorporate advanced technologies that enable more precise delivery of therapy [73]. This includes multipolar leads for optimal pacing site selection, adaptive algorithms to dynamically adjust pacing parameters based on patient-specific needs, and enhanced diagnostic capabilities to monitor heart function and therapy effectiveness over time [74]. Enhanced device technology has revolutionized the management of HF, offering advanced solutions that improve patient outcomes and quality of life [75]. These technological innovations encompass a range of devices tailored to monitor, support, and treat HF patients effectively [76].

#### 5.1.1 Cardiac Resynchronization Therapy (CRT) Devices

CRT devices have evolved significantly with enhanced features aimed at optimizing cardiac function in patients with HF [77]. Modern CRT devices utilize multipolar leads that allow for more precise pacing site selection within the heart, thereby improving electrical synchronization and enhancing therapeutic efficacy [78]. Adaptive algorithms incorporated into these devices dynamically adjust pacing parameters based on real-time assessments of cardiac function, ensuring optimal therapy delivery [79].

#### 5.1.2 Implantable Cardioverter-defibrillators (ICDs)

ICDs play a crucial role in managing HF by detecting and terminating life-threatening arrhythmias [80]. Advanced ICD technology includes faster detection algorithms to promptly identify arrhythmias, customizable programming options to tailor therapy based on individual patient needs, and enhanced battery longevity to minimize the need for frequent device replacements [80].

#### 5.1.3 Left Ventricular Assist Devices (LVADs)

LVADs provide constructed circulatory aid to patients with severe heart failure undergoing heart transplantation or as an alternative to treatment [81]. Enhanced LVAD technology features smaller device profiles, quicker operation, and improved durability, making long-term support more feasible for eligible patients [82]. Continuous flow LVADs, in particular, offer superior hemodynamic stability and reduced complications compared to earlier-generation pulsatile devices [83].

#### 5.1.4 Remote Monitoring and Telemedicine Solutions

The integration of remote monitoring and telemedicine solutions has transformed HF management by enabling proactive patient care and early intervention [84]. These technologies allow healthcare providers to remotely monitor device function, vital signs, and symptoms of HF exacerbation. Real-time data transmission facilitates timely adjustments to medication regimens, device settings, and clinical management strategies, as shown in Table **2**, thereby reducing hospitalizations and improving patient outcomes [85].

#### 5.1.5 Biocompatible Materials and Wireless Connectivity

Advancements in device materials focus on enhancing biocompatibility and reducing the risk of adverse reactions or infections. Wireless connectivity enables seamless data transmission between devices and healthcare providers, promoting continuous monitoring and proactive management of HF patients in both outpatient and home settings. These enhanced device technologies exemplify the integration of cutting-edge engineering with clinical expertise to deliver personalized and effective care for HF patients [86]. By improving device performance, durability, and connectivity, these innovations contribute to prolonging survival, enhancing quality of life, and reducing healthcare utilization among individuals living with HF [87].

#### 5.1.6 Intermittent Fasting (IF)

IF has become a viable adjunctive approach to the treatment of HF, providing several advantages that could enhance cardiac performance and general health outcomes. Studies have shown that IF can improve metabolic functions, including lowering inflammation and increasing insulin sensitivity, which are critical in the development of heart failure. The lowering of levels of systemic inflammatory cytokines may decrease the onset of heart failure [88]. Furthermore, since obesity is a known risk factor for HF, IF may help with weight management, which is an important part of managing HF. According to research on animals, IF may also enhance cardiac function by encouraging autophagy, a cellular repair process that may lessen damage to heart muscle cells. These early results suggest that intermittent fasting may play a significant role as an adjuvant in the comprehensive therapy of HF, even though additional clinical research is required [89].

### 5.2 Combined Therapies and Adjunctive Strategies

**Fig. (4):**
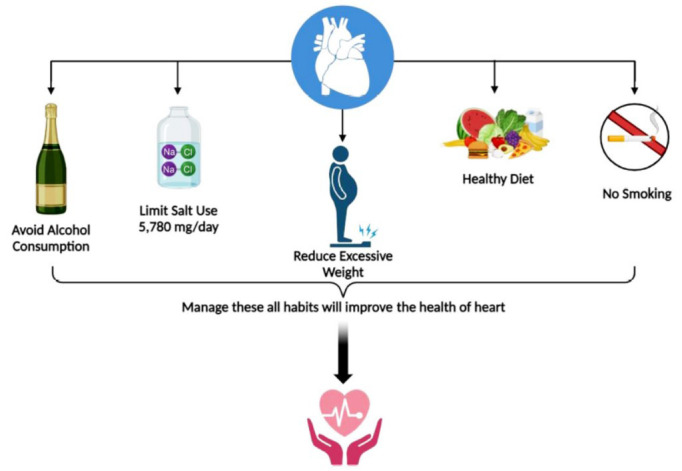
An illustration of various non-pharmacological strategies for cardiovascular (CVS) disorders. These include lifestyle modifications, such as performing regular physical activity, consuming a heart-healthy diet, smoking cessation, stress management, undergoing cardiac rehabilitation programs, controlling weight, and avoiding alcohol consumption [102].

CRT is increasingly being explored in combination with other therapies to further enhance its efficacy [90]. This includes the use of CRT in conjunction with implantable cardioverter-defibrillators to provide both resynchronization and defibrillation capabilities, as well as exploring adjunctive pharmacological therapies that may synergistically improve heart function and clinical outcomes. Adjunctive pharmacological therapies are explored to complement standard treatments and target specific aspects of HF management [91]. This includes the use of SGLT2 inhibitors, originally developed for diabetes management, which have shown additional benefits in HF by reducing cardiovascular events and hospitalizations independent of their glucose-lowering effects [92]. Similarly, novel therapies targeting myocardial energetics, inflammation pathways, or fibrosis modulation are under investigation to further optimize treatment outcomes [93]. These new opportunities for CRT underscore a paradigm shift towards personalized, evidence-based care aimed at improving outcomes and quality of life for HF patients [94]. By leveraging advanced device technologies, refining patient selection criteria, exploring combined therapies, embracing digital health solutions, and expanding research frontiers, CRT continues to evolve as a cornerstone therapy in modern HF management [95]. Comprehensive HF management incorporates lifestyle interventions, such as dietary sodium restriction, fluid management, regular physical activity, and smoking cessation [96]. Patient education plays a pivotal role in promoting adherence to these lifestyle modifications, which are essential for managing comorbidities and optimizing cardiovascular health alongside pharmacological and device-based therapies [97]. In clinical practice, combined medical therapies typically involve the simultaneous or sequential use of medications from different drug classes [98]. This strategy aims to target multiple aspects of a disease process, such as different biochemical pathways or physiological mechanisms, thereby achieving a synergistic therapeutic effect [99]. For example, in the treatment of drug-resistant epilepsy, combining traditional antiepileptic drugs with newer agents or adjuvant therapies, like the ketogenic diet or neurostimulation techniques, can improve seizure control where monotherapy fails [100]. Adjunctive advanced strategies encompass a broader spectrum of approaches beyond traditional pharmacotherapy (Fig. **4**) [101, 102]. These may include the following:

#### 5.2.1 Nutritional and Dietary Interventions

These interventions include incorporating specialized diets, like ketogenic or modified Atkins diets, alongside medication to manage conditions, such as epilepsy or metabolic disorders [103].

#### 5.2.2 Behavioral and Psychological Therapies

Combining medication with cognitive-behavioral therapy or mindfulness-based interventions can help treat conditions, such as depression, anxiety, or chronic pain [104].

#### 5.2.3 Complementary and Alternative Medicine

Integrating therapies, like acupuncture, herbal medicine, or yoga, with conventional treatments can help address symptoms and improve overall well-being [105].

#### 5.2.4 Precision Medicine Approaches

Genetic testing and personalized treatment plans can be utilized to tailor medication choices based on individual genetic profiles, thereby optimizing therapeutic outcomes and minimizing adverse reactions [106].

#### 5.2.5 Technological Interventions

Incorporating the use of advanced medical devices, such as neurostimulators, implantable pumps, or wearable health monitors, can help enhance treatment delivery and monitoring in conditions, like Parkinson's disease or chronic pain management [107].

## 6 MODULATION OF CARDIAC CONTRACTILITY IN THE TREATMENT OF HF

Cardiac contractility modulation (CCM) provides a novel method in the treatment of heart failure, aimed at improving cardiac function and symptoms in patients with chronic HF despite optimal medical therapy [108]. Recent research studies using various animal models to discover treatments for HF are shown in Table **4**. This therapy involves the delivery of electrical signals to the heart during the absolute refractory period of the cardiac cycle, enhancing myocardial contractility without causing myocardial depolarization or affecting the heart rate [109, 110].

### 6.1 Mechanism of Action

CCM operates by delivering high-energy electrical impulses to the heart during the absolute refractory period [111]. These signals are designed to modulate cardiac contractility by enhancing calcium handling within myocardial cells, which improves myocardial efficiency and contractile function [112]. Unlike traditional cardiac pacing therapies, CCM does not alter the heart rate or induce myocardial depolarization, making it suitable for patients with conduction abnormalities or arrhythmias [113, 114]. Clinical studies have demonstrated several benefits of CCM in HF management. It has been demonstrated to increase exercise tolerance, minimize symptoms, including fatigue and breathing difficulties, and enhance the quality of life of people with serious heart failure symptoms [115]. Additionally, CCM therapy has been associated with a reduction in HF hospitalizations and may improve overall survival in selected patient populations [116].

#### 6.1.1 Patient Selection Criteria

Patient selection for CCM therapy involves careful consideration of clinical criteria and device eligibility [117]. Patients with a lower left ventricular ejection fraction, symptoms of heart failure that persist during suitable medical management, and no reversible causes of HF symptoms are usually considered candidates [118]. Assessment may also include evaluation of cardiac function through echocardiography, exercise tolerance tests, and consideration of comorbidities [119].

#### 6.1.2 Device Implantation and Management

CCM therapy requires the implantation of a specialized device that delivers electrical signals to the heart. The device is typically placed *via* minimally invasive surgical techniques and involves positioning electrodes in the right ventricle. Programming of the device parameters is tailored to individual patient characteristics and response to therapy, with regular follow-up visits to monitor device function and clinical status [120].

#### 6.1.3 Therapies for Heart Failure with Ejection Fraction

HF with reduced ejection fraction (HFrEF) and heart failure with preserved ejection fraction (HFpEF) are the two more general categories. The underlying pathophysiology diagnostic standards and treatment modalities of these subgroups vary. Both HFpEF and HFrEF may benefit from improved treatment approaches thanks to personalized medicine, which uses genetic, molecular, and imaging markers. An outline of the current approaches to treating these problems in both groups is provided below:

##### 6.1.3.1 Preserved Ejection Fraction (HFpEF)

HFpEF, characterized by an ejection fraction ≥50%, is a complex syndrome with diastolic dysfunction and systemic comorbidities. Treatment strategies focus on symptom relief, comorbidity management, and improving the quality of life. Various approaches are beneficial in HFpEF, such as SGLT-2 inhibitors, MRA therapy, management of comorbidities, *etc*.

##### 6.1.3.2 Reduced Ejection Fraction (HFrEF)

HFrEF, defined by an ejection fraction ≤40%, involves systolic dysfunction and is characterized by a well-established treatment framework to reduce mortality and hospitalizations. Some examples of managing HFrEF include guideline-directed medical therapy (GDMT), angiotensin receptor-neprilysin inhibitors (ARNIs), beta-blockers, SGLT-2 inhibitors, *etc*.

#### 6.1.4 Future Directions

Ongoing research continues to explore the potential applications and refinements of CCM therapy. These include investigating its efficacy in specific HF phenotypes, optimizing device design and programming algorithms, and evaluating long-term outcomes, such as durability of therapeutic benefits and impact on overall survival. Additionally, combination therapies involving CCM with other modalities, such as pharmacological agents or device-based interventions, are being explored to further enhance treatment outcomes [121].

## CONCLUSION

In conclusion, the landscape of HF treatment has been transformed by significant advancements in device technology and therapeutic strategies. These innovations have revolutionized patient care, offering new avenues to improve outcomes and quality of life for individuals living with HF. Advanced cardiac devices, such as CRT, ICDs, and LVADs, have provided critical support for patients at various stages of HF. CRT devices, due to their ability to optimize cardiac synchronization, and ICDs, which prevent sudden cardiac death through timely arrhythmia detection and intervention, have become indispensable in managing the complexities of HF. Furthermore, LVADs have emerged as vital options for patients awaiting heart transplantation or as long-term solutions for those ineligible for transplant, offering improved durability and reducing complications compared to earlier generations. These devices not only extend survival, but also enhance the quality of life by improving exercise tolerance and reducing symptoms. Beyond device-based therapies, the integration of remote monitoring and telemedicine solutions has empowered healthcare providers to deliver personalized care, monitor patient health remotely, and intervene proactively, to prevent disease progression. This approach has significantly reduced hospitalizations, optimized medication management, and improved patient adherence to treatment regimens. Innovative pharmacological therapies, such as cardiac CCM and SGLT2 inhibitors, have further expanded treatment options, addressed specific aspects of HF pathophysiology, and offered additional benefits in symptom relief and mortality reduction. The integration of cutting-edge technologies and evidence-based therapies represents a cornerstone in modern HF management. These advancements not only improve survival rates and reduce healthcare utilization, but also empower patients to lead fuller, healthier lives. As research continues to evolve, the future holds promising prospects for continued innovation and advancement in the field of HF treatment. This paper summarizes the transformative impact of new devices and therapies on HF management while emphasizing ongoing advancements and the potential for future improvements in patient care. CCM represents a promising therapeutic approach in the management of HF, offering significant benefits in symptom relief, quality of life improvement, and potential survival for eligible patients. As research and clinical experience advance, CCM therapy continues to evolve as a valuable addition to the treatments available for HF patients.

## Figures and Tables

**Fig. (1):**
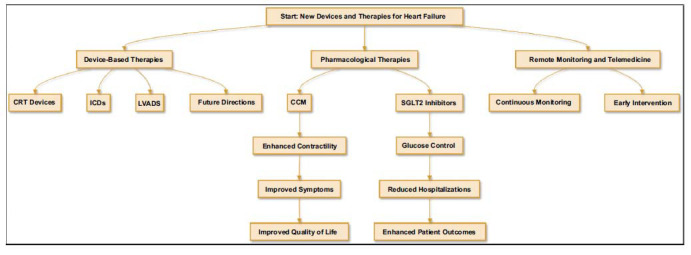
Classification of new therapeutic approaches for the management of HF. Emerging therapeutic strategies are categorized into pharmacological and device-based therapies. The pharmacological category includes advancements, such as nonsteroidal MRAs, SGLT-2 inhibitors, and dual GIP-GLP-1 receptor agonists [9].

**Fig. (2):**
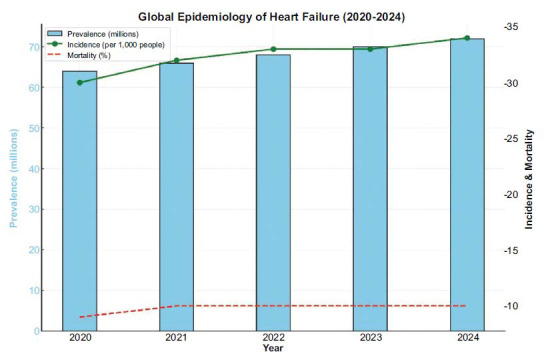
Global epidemiology of HF.

**Fig. (3):**
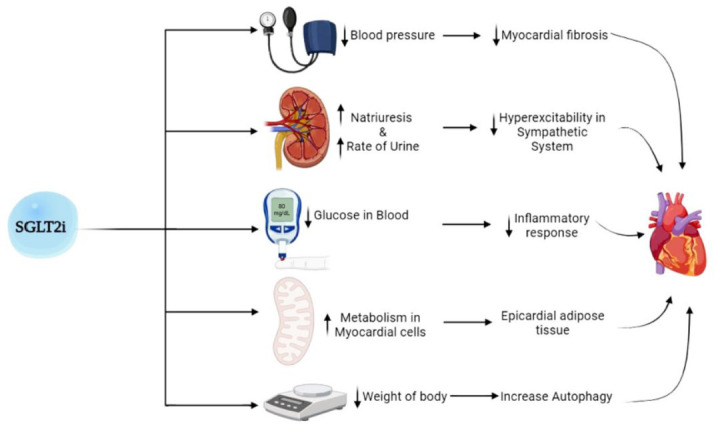
A schematic representation of the multifaceted cardiovascular benefits of sodium-glucose cotransporter-2 (SGLT2) inhibitors. Key effects include a reduction in myocardial workload through improved diuresis and natriuresis, enhancement of cardiac energy metabolism *via* ketogenesis, attenuation of inflammation and oxidative stress, and prevention of adverse cardiac remodeling.

**Table 1:** The largest cell treatment trial to date in patients with chronic HF due to low ejection fraction.

**S. No.**	**Trial Name**	**Study Details**	**Key Findings**	**Clinical Implications**	**References**
1	CONCERT- HF trial	Multicentre, randomized, controlled trial evaluating cardiac stem cell therapy in patients with chronic HF and reduced ejection fraction (rEF ≤ 40%)	- Improved function of the left ventricular chamber, reduced adverse cardiac events	Represented a milestone in cell therapy for HF, suggesting potential therapeutic benefits	[36]
2	CAREMI trial	Phase IIb randomized trial assessing intramyocardial delivery of allogeneic mesenchymal precursor cells (MPCs) in patients with ischemic HF and reduced EF (EF ≤ 40%)	- Enhanced myocardial recovery, improved exercise capacity	Demonstrated safety and efficacy of MPCs in improving heart function and quality of life	[37]
3	DREAM-HF trial	A large-scale trial investigating the use of cardio-sphere-derived cells (CDCs) in patients with HF and reduced EF (EF ≤ 35%)	- Reduced scar tissue, improved cardiac function, and exercise tolerance	Supported the role of CDCs in regenerating damaged heart tissue and improving heart function	[38]
4	ATHENA trial	Phase II trial evaluating autologous bone marrow-derived MSCs in patients with chronic HF and reduced EF (EF ≤ 40%)	- Enhanced left ventricular function, decreased HF symptoms	Highlighted MSC therapy as a potential treatment option for HF patients	[39]
5	REGENERATE-DCM trial	Examining whether autologous cardiac progenitor cells (CPCs) delivered intracoronarily are effective in treating individuals with dilated cardiomyopathy and low EF (EF < 35%)	- Improved cardiac function, decreased HF hospitalizations	Indicated that CPC therapy may be beneficial in improving outcomes in dilated cardiomyopathy	[40]

**Table 2:** Clinical evidence of HF following curable therapies in HF.

S. No.	Medication Class	Medications	Clinical Evidence	References
1	ACE inhibitors	Enalapril, lisinopril, ramipril	- Reduce mortality and hospitalization, improve symptoms and exercise tolerance	[44]
2	Angiotensin receptor blockers	Losartan, valsartan	- Similar benefits to ACE inhibitors in HF management	[45]
3	Beta-blockers	Carvedilol, metoprolol	- Reduce mortality and hospitalization, improve left ventricular function and symptoms	[46]
4	Mineralocorticoid receptor antagonists	Spironolactone, eplerenone	- Reduce mortality and hospitalization, improve symptoms, and reduce the progression of HF	[47]
5	Sacubitril/valsartan (ARNI)	Entresto	- Reduce mortality compared to ACE inhibitors alone, improve symptoms and functional status	[48]
6	SGLT2 inhibitors	Empagliflozin, dapagliflozin	- Reduce HF hospitalization, improve symptoms, and reduce renal complications	[49]
7	Ivabradine	Procoralan	- Reduce hospitalization for worsening HF symptoms	[50]

**Table 3:** Ancient ayurvedic medications used in the treatment of HF.

S. No.	Ayurvedic Medicine	Description	Mechanism of Action	Clinical Evidence	References
1	Arjuna (*Terminalia arjuna*)	Bark extract is used for cardiovascular support	Strengthens cardiac muscle, and improves heart function	Studies suggest improved left ventricular function and exercise capacity	[65]
2	Guggul (*Commiphora mukul*)	Resin extract is known for lipid-lowering and anti-inflammatory properties	Reduces cholesterol levels, and has anti-inflammatory effects	Improves lipid profile and may reduce the risk factors of heart disease	[66]
3	Garlic (*Allium sativum*)	Used for cardiovascular health, including blood pressure regulation	Vasodilation, antiplatelet effects, antioxidant properties	May lower blood pressure and reduce cardiovascular risk	[67]
4	Hawthorn (*Crataegus* spp.)	Berries and leaves are used for heart health, including mild HF	Improves coronary artery blood flow, and strengthens heart contractions	Shows promise in improving symptoms and exercise tolerance in HF	[68]
5	*Terminalia chebula* (Haritaki)	Fruit is used in Ayurvedic formulations for digestive and cardiovascular health	Antioxidant and anti-inflammatory effects, and supports heart function	Limited clinical evidence; traditionally used for general health support	[69]
6	Amla (*Emblica officinalis*)	Rich in vitamin C, used for cardiovascular support and antioxidant properties	Anti-inflammatory effects and reduces oxidative stress	Supports heart health and may reduce the risk factors of heart disease	[70]

**Table 4:** Recent studies using animal models to explore treatments for HF.

Study	Animal Model	Therapeutic Approaches	Clinical Implications	References
Stem cell therapy	Rat model of myocardial infarction	Intramyocardial injection of cardiac progenitor cells (CPCs)	Demonstrates the potential of CPCs in regenerating heart tissue and improving function post-infarction	[122]
Gene therapy	Canine model of dilated cardiomyopathy	Adeno-associated virus vector delivery of SERCA2a gene	Shows promise in gene therapy for correcting calcium dysregulation in dilated cardiomyopathy	[123]
Pharmacological therapy	Porcine model of HF	Administration of novel beta3-adrenergic receptor agonist	Highlights potential of beta3-agonists as therapeutic agents in HF management	[124, 125]
Device therapy	Murine model of HF	Implantation of CCM devices	Suggests CCM device efficacy in enhancing heart function and performance in murine models	[126]
Nanotechnology	Rabbit model of ischemic heart disease	Delivery of nanocarriers loaded with growth factors	Indicates the potential of nanotechnology in targeted delivery for heart disease treatment	[127, 128]

## LIST OF ABBREVIATIONS

ACE: Angiotensin-converting Enzyme
CCM: Cardiac Contractility Modulation
CRT: Cardiac Resynchronization Therapy
CVD: Cardiovascular Disease
HFpEF: Heart Failure with Preserved Ejection Fraction
HFrEF: Heart Failure with Reduced Ejection Fraction
HF: Heart Failure
ICD: Implantable Cardioverter-defibrillator
LVAD: Left Ventricular Assist Device
SGLT2: Sodium-glucose Co-transporter-2

## References

[1] Andreeva E.R., Pugach I.M., Orekhov A.N. (1997). Subendothelial smooth muscle cells of human aorta express macrophage antigen *in situ* and in vitro.. Atherosclerosis.

[2] Aujesky D., Obrosky D.S., Stone R.A. (2005). Derivation and validation of a prognostic model for pulmonary embolism.. Am. J. Respir. Crit. Care Med..

[3] Aune D, Sen A, ó’Hartaigh B (2017). Resting heart rate and the risk of cardiovascular disease, total cancer, and all-cause mortality – A systematic review and dose–response meta-analysis of prospective studies.. Nutr. Metab. Cardiovasc. Dis..

[4] Bainbridge M.N., Li L., Tan Y., Cheong B.Y., Marian A.J. (2017). Identification of established arrhythmogenic right ventricular cardiomyopathy mutation in a patient with the contrasting phenotype of hypertrophic cardiomyopathy.. BMC Med. Genet..

[5] Basili S., Raparelli V., Vestri A., Tanna G.L.D., Violi F. (2010). Comparison of efficacy of antiplatelet treatments for patients with claudication.. Thromb. Haemost..

[6] Basman C., Rashid U., Parmar Y.J., Kliger C., Kronzon I. (2018). The role of percutaneous vacuum-assisted thrombectomy for intracardiac and intravascular pathology.. J. Card. Surg..

[7] Bauriedel G., Hutter R., Welsch U., Bach R., Sievert H., Lüderitz B. (1999). Role of smooth muscle cell death in advanced coronary primary lesions: implications for plaque instability.. Cardiovasc. Res..

[8] Beebe H.G., Dawson D.L., Cutler B.S. (1999). A new pharmacological treatment for intermittent claudication: results of a randomized, multicenter trial.. Arch. Intern. Med..

[9] Berger J.S., Abramson B.L., Lopes R.D. (2018). Ticagrelor versus clopidogrel in patients with symptomatic peripheral artery disease and prior coronary artery disease: Insights from the EUCLID trial.. Vasc. Med..

[10] Belch J.J.F., Dormandy J., Biasi G.M. (2010). Results of the randomized, placebo-controlled clopidogrel and acetylsalicylic acid in bypass surgery for peripheral arterial disease (CASPAR) trial.. J. Vasc. Surg..

[11] Bennett M.R., Sinha S., Owens G.K. (2016). Vascular smooth muscle cells in atherosclerosis.. Circ. Res..

[12] Berger J., Heizer G., Baumgartner I. (2017). Ticagrelor in patientswtih symptomatic peripheral artery disease and prior coronary artery disease.. Eur. Heart J..

[13] Bergqvis D. (1994). Platelet inhibition with ASA/dipyridamole after percutaneous balloon angioplasty in patients with symptomatic lower limb arterial disease. A prospective double-blind trial.. Eur. J. Vasc. Surg..

[14] Bhatt A.S., DeVore A.D., DeWald T.A., Swedberg K., Mentz R.J. (2017). Achieving a maximally tolerated β-blocker dose in heart failure patients.. J. Am. Coll. Cardiol..

[15] Bhatt D.L., Flather M.D., Hacke W. (2007). Patients with prior myocardial infarction, stroke, or symptomatic peripheral arterial disease in the CHARISMA trial.. J. Am. Coll. Cardiol..

[16] Bhonsale A., James C.A., Tichnell C. (2013). Risk stratification in arrhythmogenic right ventricular dysplasia/cardiomyopathy-associated desmosomal mutation carriers.. Circ. Arrhythm. Electrophysiol..

[17] Blanchard J., Carreras L.O., Kindermans M. (1994). Results of EMATAP:] A double-blind placebo-controlled multicentre trial of ticlopidine in patients with peripheral arterial disease.. Nouv. Rev. Fr. Hematol..

[18] Bocchi E.A., Böhm M., Borer J.S. (2015). Effect of combining ivabradine and β-blockers: Focus on the use of carvedilol in the shift population.. Cardiology.

[19] Bonaca M.P., Nault P., Giugliano R.P. (2018). Low-density lipoprotein cholesterol lowering with evolocumab and outcomes in patients with peripheral artery disease.. Circulation.

[20] Borer J.S., Böhm M., Ford I. (2012). Effect of ivabradine on recurrent hospitalization for worsening heart failure in patients with chronic systolic heart failure: The SHIFT study.. Eur. Heart J..

[21] Büller H.R., Prins M.H., Lensin A.W. (2012). Oral rivaroxaban for the treatment of symptomatic pulmonary embolism.. N. Engl. J. Med..

[22] Caldwell J., Redfearn D., Chiale P.A., Baranchuk A. (2013). Ablation-induced epsilon wave.. Heart Rhythm.

[23] Chang J.R., Duan X.H., Zhang B.H. (2013). Intermedin1-53 attenuates vascular smooth muscle cell calcification by inhibiting endoplasmic reticulum stress *via* cyclic adenosine monophosphate/protein kinase A pathway.. Exp. Biol. Med. (Maywood).

[24] Chatterjee S., Chakraborty A., Weinberg I. (2014). Thrombolysis for pulmonary embolism and risk of all-cause mortality, major bleeding, and intracranial hemorrhage: A meta-analysis.. JAMA.

[25] Chauhan M.S., Kawamura A. (2007). Percutaneous rheolytic thrombectomy for large pulmonary embolism: A promising treatment option.. Catheter. Cardiovasc. Interv..

[26] Clarke M.C.H., Figg N., Maguire J.J. (2006). Apoptosis of vascular smooth muscle cells induces features of plaque vulnerability in atherosclerosis.. Nat. Med..

[27] Croons V., Martinet W., Herman A.G., De Meyer G.R.Y. (2008). Differential effect of the protein synthesis inhibitors puromycin and cycloheximide on vascular smooth muscle cell viability.. J. Pharmacol. Exp. Ther..

[28] Daly C.A., Clemens F., Lopez Sendon J.L. (2010). Inadequate control of heart rate in patients with stable angina: Results from the European Heart Survey.. Postgrad. Med. J..

[29] Donaldson C.W., Baker J.N., Narayan R.L. (2015). Thrombectomy using suction filtration and veno‐venous bypass: Single center experience with a novel device.. Catheter. Cardiovasc. Interv..

[30] Gal G., Fine M.J., Roy P-M. (2008). Prospective validation of the Pulmonary Embolism Severity Index. A clinical prognostic model for pulmonary embolism.. Thromb. Haemost..

[31] Dumantepe M., Teymen B., Akturk U., Seren M. (2015). Efficacy of rotational thrombectomy on the mortality of patients with massive and submassive pulmonary embolism.. J. Card. Surg..

[32] Dzau V.J., Braun-Dullaeus R.C., Sedding D.G. (2002). Vascular proliferation and atherosclerosis: New perspectives and therapeutic strategies.. Nat. Med..

[33] Fasullo S., Scalzo S., Maringhini G. (2011). Six-month echocardiographic study in patients with submassive pulmonary embolism and right ventricle dysfunction: comparison of thrombolysis with heparin.. Am. J. Med. Sci..

[34] Ferrari R., Fox K. (2016). Heart rate reduction in coronary artery disease and heart failure.. Nat. Rev. Cardiol..

[35] Fontaine G., Fontaliran F., Hébert J.L. (1999). Arrhythmogenic right ventricular dysplasia.. Annu. Rev. Med..

[36] Fontaine G., Fontaliran F. (1999). Arrhythmogenic right ventricular dysplasia masquerading as dilated cardiomyopathy.. Am. J. Cardiol..

[37] Fontaine G., Guiraudon G., Frank R. (1977). Simulation studies and epicardial mapping in ventricular tachycardia: Study of mechanisms and selection for surgery. Re-entrant Arrhythmias Mechanisms and Treatment..

[38] Fox K., Ford I., Steg P.G., Tardif J.C., Tendera M., Ferrari R. (2014). Ivabradine in stable coronary artery disease without clinical heart failure.. N. Engl. J. Med..

[39] Fox K., Ford I., Steg P.G., Tendera M., Robertson M., Ferrari R. (2008). Heart rate as a prognostic risk factor in patients with coronary artery disease and left-ventricular systolic dysfunction (BEAUTIFUL): A subgroup analysis of a randomised controlled trial.. Lancet.

[40] George B.A., Ko J.M., Lensing F.D., Kuiper J.J., Roberts W.C. (2011). “Repaired” tetralogy of fallot mimicking arrhythmogenic right ventricular cardiomyopathy (another phenocopy).. Am. J. Cardiol..

[41] Giavarini A., de Silva R. (2016). The role of ivabradine in the management of angina pectoris.. Cardiovasc. Drugs Ther..

[42] Gottschalk B., Gysel M., Barbosa-Barros R. (2014). The use of fontaine leads in the diagnosis of arrhythmogenic right ventricular dysplasia.. Ann. Noninvasive Electrocardiol..

[43] Habal M.V., Liu P.P., Austin P.C. (2014). Association of heart rate at hospital discharge with mortality and hospitalizations in patients with heart failure.. Circ. Heart Fail..

[44] Hamill V., Ford I., Fox K. (2015). Repeated heart rate measurement and cardiovascular outcomes in left ventricular systolic dysfunction.. Am. J. Med..

[45] Heidenreich P.A., Bozkurt B., Aguilar D. (2022). 2022 AHA/ACC/HFSA guideline for the management of heart failure: A report of the American College of Cardiology/American Heart Association Joint Committee on clinical practice guidelines.. Circulation.

[46] Henderson E.L., Geng Y.J., Sukhova G.K., Whittemore A.D., Knox J., Libby P. (1999). Death of smooth muscle cells and expression of mediators of apoptosis by T lymphocytes in human abdominal aortic aneurysms.. Circulation.

[47] Hiatt W. (2017). A study comparing cardiovascular effects of ticagrelor and clopidogrel in patients with peripheral artery disease (EUCLID)..

[48] Hiatt W.R., Money S.R., Brass E.P. (2008). Long-term safety of cilostazol in patients with peripheral artery disease: The CASTLE study (Cilostazol: A Study in Long-term Effects).. J. Vasc. Surg..

[49] Hoogendijk M.G. (2012). Diagnostic dilemmas: Overlapping features of brugada syndrome and arrhythmogenic right ventricular cardiomyopathy.. Front. Physiol..

[50] Hurst J.W. (1998). Naming of the waves in the ECG, with a brief account of their genesis.. Circulation.

[51] Fudim M., Abraham W.T., von Bardeleben R.S. (2021). Device therapy in chronic heart failure.. J. Am. Coll. Cardiol..

[52] Ibrahim N.E., Gaggin H.K., Turchin A. (2019). Heart rate, beta-blocker use, and outcomes of heart failure with reduced ejection fraction.. Eur. Heart J. Cardiovasc. Pharmacother..

[53] Iida O., Yokoi H., Soga Y. (2013). Cilostazol reduces angiographic restenosis after endovascular therapy for femoropopliteal lesions in the sufficient treatment of peripheral intervention by cilostazol study.. Circulation.

[54] Iyemere V.P., Proudfoot D., Weissberg P.L., Shanahan C.M. (2006). Vascular smooth muscle cell phenotypic plasticity and the regulation of vascular calcification.. J. Intern. Med..

[55] Janzon L., Bergqvist D., Boberg J. (1990). Prevention of myocardial infarction and stroke in patients with intermittent claudication; effects of ticlopidine. Results from STIMS, the Swedish Ticlopidine Multicentre Study.. J. Intern. Med..

[56] Jaoude S.A., Leclercq J.F., Coumel P. (1996). Progressive ECG changes in arrhythmogenic right ventricular disease Evidence foran evolving disease.. Eur. Heart J..

[57] Jiménez D., Aujesky D., Moores L. (2010). Simplification of the pulmonary embolism severity index for prognostication in patients with acute symptomatic pulmonary embolism.. Arch. Intern. Med..

[58] Jones W.S., Baumgartner I., Hiatt W.R. (2017). International steeringcommittee and investigators of the euclid trial. ticagrelorcompared with clopidogrel in patients with prior lower extremity revascularization for peripheral artery disease.. Circulation.

[59] Jukema J.W., Szarek M., Zijlstra L.E. (2019). Alirocumab in patients with polyvascular disease and recent acute coronary syndrome.. J. Am. Coll. Cardiol..

[60] Kapustin A.N., Shanahan C.M. (2012). Calcium regulation of vascular smooth muscle cell-derived matrix vesicles.. Trends Cardiovasc. Med..

[61] Karsenty G., Kronenberg H.M., Settembre C. (2009). Genetic control of bone formation.. Annu. Rev. Cell Dev. Biol..

[62] Kaski J.C., Gloekler S., Ferrari R. (2018). Role of ivabradine in management of stable angina in patients with different clinical profiles.. Open Heart.

[63] Katsanos K., Spiliopoulos S., Saha P. (2015). Comparative efficacy and safety of different antiplatelet agents for prevention of major cardiovascular events and leg amputations in patients with peripheral arterial disease: A systematic review and network meta-analysis.. PLoS One.

[64] Kau L.U., Natrajan S., Dalal J., Saran R.K. (2017). Prevalence and control of cardiovascular risk factors in stable coronary artery outpatients in India compared with the rest of the world: An analysis from international CLARIFY registry.. Indian Heart J..

[65] Kaul U., Wander G.S., Sinha N. (2020). Self-blood pressure measurement as compared to office blood pressure measurement in a large Indian population; the India Heart Study.. J. Hypertens..

[66] Kedi X., Ming Y., Yongping W., Yi Y., Xiaoxiang Z. (2009). Free cholesterol overloading induced smooth muscle cells death and activated both ER- and mitochondrial-dependent death pathway.. Atherosclerosis.

[67] Kenigsberg D.N., Kalahasty G., Grizzard J.D., Wood M.A., Ellenbogen K.A. (2007). Images in cardiovascular medicine. Intracardiac correlate of the epsilon wave in a patient with arrhythmogenic right ventricular dysplasia.. Circulation.

[68] Khan M.I., Pichna B.A., Shi Y., Bowes A.J., Werstuck G.H. (2009). Evidence supporting a role for endoplasmic reticulum stress in the development of atherosclerosis in a hyperglycaemic mouse model.. Antioxid. Redox Signal..

[69] Kiès P., Bootsma M., Bax J.J. (2006). Serial reevaluation for ARVD/C is indicated in patients presenting with left bundle branch block ventricular tachycardia and minor ECG abnormalities.. J. Cardiovasc. Electrophysiol..

[70] Komajda M., Tavazzi L., Swedberg K. (2016). Chronic exposure to ivabradine reduces readmissions in the vulnerable phase after hospitalization for worsening systolic heart failure: A post‐hoc analysis of SHIFT.. Eur. J. Heart Fail..

[71] Konstantinides S., Geibel A., Olschewski M. (1997). Association between thrombolytic treatment and the prognosis of hemodynamically stable patients with major pulmonary embolism: Results of a multicenter registry.. Circulation.

[72] Konstantinides S., Tiede N., Geibel A., Olschewski M., Just H., Kasper W. (1998). Comparison of alteplase versus heparin for resolution of major pulmonary embolism.. Am. J. Cardiol..

[73] Konstantinides S.V., Meyer G., Becattini C. (2019). 2019 ESC Guidelines for the diagnosis and management of acute pulmonary embolism developed in collaboration with the European Respiratory Society (ERS).. Eur. Respir. J..

[74] Koruth J.S., Lala A., Pinney S., Reddy V.Y., Dukkipati S.R. (2017). The clinical use of ivabradine.. J. Am. Coll. Cardiol..

[75] Kurgansky K.E., Schubert P., Parker R. (2020). Association of pulse rate with outcomes in heart failure with reduced ejection fraction: A retrospective cohort study.. BMC Cardiovasc. Disord..

[76] Lam C.S.P., Teng T.H.K., Tay W.T. (2016). Regional and ethnic differences among patients with heart failure in Asia: The Asian sudden cardiac death in heart failure registry.. Eur. Heart J..

[77] Larroque-Cardoso P., Swiader A., Ingueneau C. (2013). Role of protein kinase C δ in ER stress and apoptosis induced by oxidized LDL in human vascular smooth muscle cells.. Cell Death Dis..

[78] Letsas K.P., Efremidis M., Weber R. (2011). Epsilon-like waves and ventricular conduction abnormalities in subjects with type 1 ECG pattern of Brugada syndrome.. Heart Rhythm.

[79] Li G.L., Saguner A.M., Fontaine G.H., Frank R. (2018). Epsilon waves: Milestones in the discovery and progress.. Ann. Noninvasive Electrocardiol..

[80] Libby P., Ridker P.M., Hansson G.K. (2011). Progress and challenges in translating the biology of atherosclerosis.. Nature.

[81] Liberman M., Johnson R.C., Handy D.E., Loscalzo J., Leopold J.A. (2011). Bone morphogenetic protein-2 activates NADPH oxidase to increase endoplasmic reticulum stress and human coronary artery smooth muscle cell calcification.. Biochem. Biophys. Res. Commun..

[82] Link M.S., Laidlaw D., Polonsky B. (2014). Ventricular arrhythmias in the North American multidisciplinary study of ARVC: Predictors, characteristics, and treatment.. J. Am. Coll. Cardiol..

[83] Liu H., Li X., Qin F., Huang K. (2014). Selenium suppresses oxidative-stress-enhanced vascular smooth muscle cell calcification by inhibiting the activation of the PI3K/AKT and ERK signaling pathways and endoplasmic reticulum stress.. J. Biol. Inorg. Chem..

[84] Liu X., Johnson M., Mardekian J., Phatak H., Thompson J., Cohen A.T. (2015). Apixaban reduces hospitalizations in patients with venous thromboembolism: An analysis of the apixaban for the initial management of pulmonary embolism and deep-vein thrombosis as first-line therapy (AMPLIFY) trial.. J. Am. Heart Assoc..

[85] Liu Y., Drozdov I., Shroff R., Beltran L.E., Shanahan C.M. (2013). Prelamin A accelerates vascular calcification *via* activation of the DNA damage response and senescence-associated secretory phenotype in vascular smooth muscle cells.. Circ. Res..

[86] London G.M. (2011). Arterial calcification: Cardiovascular function and clinical outcome.. Nefrología.

[87] Lu Y., Bian Y., Wang Y., Bai R., Wang J., Xiao C. (2015). Globular adiponectin reduces vascular calcification *via* inhibition of ER-stress-mediated smooth muscle cell apoptosis.. Int. J. Clin. Exp. Pathol..

[88] Maddox T.M., Januzzi J.L., Allen L.A. (2021). 2021 update to the 2017 ACC expert consensus decision pathway for optimization of heart failure treatment: Answers to 10 pivotal issues about heart failure with reduced ejection fraction.. J. Am. Coll. Cardiol..

[89] Malabanan K.P., Kanellakis P., Bobik A., Khachigian L.M. (2008). Activation transcription factor-4 induced by fibroblast growth factor-2 regulates vascular endothelial growth factor-A transcription in vascular smooth muscle cells and mediates intimal thickening in rat arteries following balloon injury.. Circ. Res..

[90] Masuda M., Miyazaki-Anzai S., Keenan A.L. (2015). Saturated phosphatidic acids mediate saturated fatty acid–induced vascular calcification and lipotoxicity.. J. Clin. Invest..

[91] Masuda M., Ting T.C., Levi M., Saunders S.J., Miyazaki-Anzai S., Miyazaki M. (2012). Activating transcription factor 4 regulates stearate-induced vascular calcification.. J. Lipid Res..

[92] McDonagh T.A., Metra M., Adamo M. (2021). 2021 ESC Guidelines for the diagnosis and treatment of acute and chronic heart failure.. Eur. Heart J..

[93] Mullasari A., Mahajan A., Chanana B.B. (2020). Efficacy and safety of ivabradine once-daily prolonged-release versus twice-daily immediate- release formulation in patients with stable chronic heart failure with systolic dysfunction: A randomized, double-blind, phase 3 non-inferiority (PROFICIENT) study.. Cardiol. Ther..

[94] Neven E., Dauwe S., De Broe M.E., D’Haese P.C., Persy V. (2007). Endochondral bone formation is involved in media calcification in rats and in men.. Kidney Int..

[95] Padmanabhan T.N.C., Dani S., Chopra V.K., Guha S., Vasnawala H., Ammar R. (2014). Prevalence of sympathetic overactivity in hypertensive patients – A pan India, non-interventional, cross sectional study.. Indian Heart J..

[96] Peters S., Trümmel M. (2003). Diagnosis of arrhythmogenic right ventricular dysplasia-cardiomyopathy: Value of standard ECG revisited.. Ann. Noninvasive Electrocardiol..

[97] Platonov P.G., Calkins H., Hauer R.N. (2016). High interobserver variability in the assessment of epsilon waves: Implications for diagnosis of arrhythmogenic right ventricular cardiomyopathy/dysplasia.. Heart Rhythm.

[98] Platonov P.G., Haugaa K.H., Bundgaard H. (2019). Primary prevention of sudden cardiac death with implantable cardioverter-defibrillator therapy in patients with arrhythmogenic right ventricular cardiomyopathy.. Am. J. Cardiol..

[99] Proudfoot D., Shanahan C.M. (2001). Biology of calcification in vascular cells: Intima versus media.. Herz.

[100] Proudfoot D., Skepper J.N., Hegyi L., Bennett M.R., Shanahan C.M., Weissberg P.L. (2000). Apoptosis regulates human vascular calcification] in vitro: Evidence for initiation of vascular calcification by apoptotic bodies.. Circ. Res..

[101] Qiao J.H., Mertens R.B., Fishbein M.C., Geller S.A. (2003). Cartilaginous metaplasia in calcified diabetic peripheral vascular disease: Morphologic evidence of enchondral ossification.. Hum. Pathol..

[102] Santucci P, Morton J, Picken M, Wilber D (2004). Electroanatomic mapping of the right ventricle in a patient with a giant epsilon wave, ventricular tachycardia, and cardiac sarcoidosis.

[103] Rao D., Balagopalan J.P., Sharma A., Chauhan V.C., Jhala D. (2015). BEAT survey: A cross-sectional study of resting heart rate in young (18-55 year) hypertensive patients.. J. Assoc. Physicians India.

[104] Rao M.S., Mandal S. (2017). Epidemiologic surveillance on quality of life in patients with systolic heart failure after treatment with the selective heart rate inhibitor ivabradine.. J. Pract. Cardiovasc. Sci..

[105] Rennenberg R., Kessels A.G.H., Schurgers L.J., Van Engelshoven J.M.A., de Leeuw P., Kroon A.A. (2009). Vascular calcifications as a marker of increased cardiovascular risk: A meta-analysis.. Vasc. Health Risk Manag..

[106] Rong J.X., Shapiro M., Trogan E., Fisher E.A. (2003). Transdifferentiation of mouse aortic smooth muscle cells to a macrophage-like state after cholesterol loading.. Proc. Natl. Acad. Sci. USA.

[107] Sage A.P., Tintut Y., Demer L.L. (2010). Regulatory mechanisms in vascular calcification.. Nat. Rev. Cardiol..

[108] Sengupta S.P., Burkule N., Bansal M. (2021). Normative values of cardiac chamber dimensions and global longitudinal strain in Indians: The Indian Normative Data of Echocardiography Analyzed (INDEA) study.. Int. J. Cardiovasc. Imaging.

[109] Serrano R.L., Yu W., Terkeltaub R. (2014). Mono-allelic and bi-allelic ENPP1 deficiency promote post-injury neointimal hyperplasia associated with increased C/EBP homologous protein expression.. Atherosclerosis.

[110] Shanahan C.M., Cary N.R.B., Salisbury J.R., Proudfoot D., Weissberg P.L., Edmonds M.E. (1999). Medial localization of mineralization-regulating proteins in association with Mönckeberg’s sclerosis: Evidence for smooth muscle cell-mediated vascular calcification.. Circulation.

[111] Shanahan C.M., Weissberg P.L., Metcalfe J.C. (1993). Isolation of gene markers of differentiated and proliferating vascular smooth muscle cells.. Circ. Res..

[112] Shanahan C.M. (2005). Vascular calcification.. Curr. Opin. Nephrol. Hypertens..

[113] Shankman L.S., Gomez D., Cherepanova O.A. (2015). KLF4-dependent phenotypic modulation of smooth muscle cells has a key role in atherosclerotic plaque pathogenesis.. Nat. Med..

[114] Shrivastava A., Goyal M.K., Gupta J.K. (2020). Epileptogenic drugs and seizures: A comprehensive review of current knowledge.. Int J Pharm Res.

[115] Shrivastava A., Gupta J.K., Goyal M.K. (2022). Flavonoids and antiepileptic drugs: A comprehensive review on their neuroprotective potentials.. J Med P’ceutical Allied Sci.

[116] Shrivastava A., Gupta J.K., Goyal M.K. (2022). Neuroprotective efficacy of quercetin with lamotrigine and gabapentin against pentylenetetrazole- Induced kindling and associated behavioral comorbidities in mice.. Indian J Pharmaceut Educ Res.

[117] Steg P.G., Ferrari R., Ford I. (2012). Heart rate and use of beta-blockers in stable outpatients with coronary artery disease.. PLoS One.

[118] Uhl H.S. (1952). A previously undescribed congenital malformation of the heart: almost total absence of the myocardium of the right ventricle.. Bull. Johns Hopkins Hosp..

[119] Werstuck G.H., Khan M.I., Femia G. (2006). Glucosamine-induced endoplasmic reticulum dysfunction is associated with accelerated atherosclerosis in a hyperglycemic mouse model.. Diabetes.

[120] Yu J., Hu J., Dai X. (2014). SCN5A mutation in Chinese patients with arrhythmogenic right ventricular dysplasia.. Herz.

[121] Zhou A.X., Wang X., Lin C.S. (2015). C/EBP-homologous protein (CHOP) in vascular smooth muscle cells regulates their proliferation in aortic explants and atherosclerotic lesions.. Circ. Res..

[122] Estep J.D., Salah H.M., Kapadia S.R. (2024). HFSA scientific statement: Update on device based therapies in heart failure.. J. Card. Fail..

[123] McMurray J.J., Packer M., Desai A.S. (2023). Angiotensin–neprilysin inhibition versus enalapril in heart failure.. N. Engl. J. Med..

[124] Dahlström U., Jhund P.S., McMurray J.J. (2023). The role of nonsteroidal mineralocorticoid receptor antagonists (MRA) in heart failure treatment: A review.. Lancet Cardiol.

[125] Lunar IG, Blanco I (2020). Fernández-Friera l, *et al.* Design of the β3-
adrenergic agonist treatment in chronic pulmonary hypertension
secondary to heart failure trial. J Am Coll Cardiol Basic Trans Sci.

[126] Boehmer J.P., Topol E.J., Ferguson T. (2023). Advances in the use of implantable cardioverter defibrillators (ICD) for heart failure management.. J. Cardiovasc. Electrophysiol..

[127] Eid S.M., Eltawil K., Al-Kahtani M.A. (2016). Antihypertensive effects of herbal medicines: A review of recent studies.. Phytother. Res..

[128] (2017). Eid SM, El-Mesery SH. The role of Zingiber officinale and Allium sativum in the treatment of hypertension: Mechanisms and clinical evidence.. J Ethnopharmacol.

